# Bio-Mapping Indicators and Pathogen Loads in a Commercial Broiler Processing Facility Operating with High and Low Antimicrobial Intervention Levels

**DOI:** 10.3390/foods11060775

**Published:** 2022-03-08

**Authors:** Juan F. De Villena, David A. Vargas, Rossy Bueno López, Daniela R. Chávez-Velado, Diego E. Casas, Reagan L. Jiménez, Marcos X. Sanchez-Plata

**Affiliations:** International Center for Food Industry Excellence, Department of Animal and Food Sciences, Texas Tech University, Lubbock, TX 79409, USA; juan.devillena@ttu.edu (J.F.D.V.); andres.vargas@ttu.edu (D.A.V.); rossy.bueno@ttu.edu (R.B.L.); daniela.r.chavez@ttu.edu (D.R.C.-V.); diego.casas@ttu.edu (D.E.C.); reagan.brashears@ttu.edu (R.L.J.)

**Keywords:** poultry bio-mapping, chemical interventions, *Salmonella* enumeration, *Campylobacter* enumeration

## Abstract

The poultry industry in the United States has traditionally implemented non-chemical and chemical interventions against *Salmonella* spp. and *Campylobacter* spp. on the basis of experience and word-of-mouth information shared among poultry processors. The effects of individual interventions have been assessed with microbiological testing methods for *Salmonella* spp. and *Campylobacter* spp. prevalence as well as quantification of indicator organisms, such as aerobic plate counts (APC), to demonstrate efficacy. The current study evaluated the loads of both indicators and pathogens in a commercial chicken processing facility, comparing the “normal chemical”, with all chemical interventions turned-on, at typical chemical concentrations set by the processing plant versus low-chemical process (“reduced chemical”), where all interventions were turned off or reduced to the minimum concentrations considered in the facility’s HACCP system. Enumeration and prevalence of *Salmonella* spp. and *Campylobacter* spp. as well as indicator organisms (APC and Enterobacteriaceae—EB) enumeration were evaluated to compare both treatments throughout a 25-month sampling period. Ten locations were selected in the current bio-mapping study, including live receiving, rehanger, post eviscerator, post cropper, post neck breaker, post IOBW #1, post IOBW #2, prechilling, post chilling, and parts (wings). Statistical process control parameters for each location and processing schemes were developed for each pathogen and indicator evaluated. Despite demonstrating significant statistical differences between the normal and naked processes in *Salmonella* spp. counts (“normal” significantly lower counts than the “reduced” at each location except for post-eviscerator and post-cropper locations), the prevalence of *Salmonella* spp. after chilling is comparable on both treatments (~10%), whereas for *Campylobacter* spp. counts, only at the parts’ location was there significant statistical difference between the “normal chemical” and the “reduced chemical”. Therefore, not all chemical intervention locations show an overall impact on *Salmonella* spp. or *Campylobacter* spp., and certain interventions can be turned off to achieve the same or better microbial performance if strategic intervention locations are enhanced.

## 1. Introduction

The United States poultry industry is the largest producer and the second largest exporter of poultry meat in the world [1]. In 2020, the value of production combining broilers, eggs, and turkeys was USD 35.5 billion, with 61% from broilers, 24% from eggs, 15% from turkeys, and less than 1% from chickens (e.g., spent fowl) [2]. Moreover, consumption of poultry meat has been trending up in the last ten years, displacing a significant amount of red meat consumption perhaps in part because of favorable prices and health recommendations. According to the National Chicken Council, the per-capita consumption of poultry in the United States in 2020 was 113.4 lb, from which 97.6 lb were chicken, and 15.8 lb were turkey [3]. Furthermore, with almost 18% of total poultry production exported, the U.S. poultry industry is heavily influenced by currency fluctuations, trade negotiations, and economic growth in importing markets [2].

The Center for Disease Control and Prevention (CDC), in 2013, estimated that in the United States (U.S.), there are around 48 million people who suffer from foodborne illnesses every year: 128,000 required hospitalization, and 3000 died. Furthermore, the contribution of poultry and eggs to foodborne illnesses caused by bacteria is 22.8%, which is the second highest percentage overall for illnesses compared to land animals (meat: 23.2%) [4]. *Salmonella* spp. is one of the leading causes of foodborne illnesses, after Norovirus, accounting for approximately 1.1 million cases per year, with 19,336 hospitalizations and 378 deaths [5]. The CDC also notes that campylobacteriosis, caused by *Campylobacter* spp., is the most common bacterial cause of diarrheal illness in the U.S., with approximately 20 cases diagnosed annually for every 100,000 people [5]. The CDC estimates that *Campylobacter* spp. is responsible for infecting at least 1.5 million U.S. residents every year [6]. Therefore, the impact of these two pathogens on public health is a significant concern in the United States and globally [7,8].

The United States Department of Agriculture (USDA)—Food Safety and Inspection Service (FSIS) enforces microbial performance standards based on prevalence (positive or negative) in poultry-processing establishments. Whole birds and parts are collected after the chilling step, sent out to an official laboratory, and tested for *Salmonella* spp. as part of this verification system. FSIS established the *Salmonella* spp. performance standard of 5 positive results out of 51 samples collected (for whole birds) and 8 positive results out of 52 samples collected (for parts, e.g., wings). There is a *Campylobacter* spp. standard; however, it is not currently enforced. Whole-bird and/or part samples are collected one per week, and each result is entered into a 52-week moving window database that calculates individual plant performance and categorizes establishments in three categories. Category 1 is defined as establishments that have achieved 50% or less of the maximum allowable percent positive during the most recently completed 52-week moving window. Category 2 is for establishments that meet the maximum allowable percent positive but have results greater than 50% of the maximum allowable percent positive during the most recently completed 52-week moving window, and Category 3 is for establishments that have exceeded the maximum allowable percent positive during the most recently completed 52-week moving window [9]. Therefore, the focus remains in reducing the prevalence of *Salmonella* spp. through the implementation of sanitary dressing procedures, applying antimicrobial interventions, both chemical and non-chemical, to reduce cross contamination during processing and handling [10].

Most chicken processors in the U.S. proactively work to minimize pathogen contamination and comply with regulatory performance standards using process control and pathogen reduction initiatives based on Hazard Analysis and Critical Control Points (HACCP) systems to reduce consumer exposure to foodborne pathogens, such as *Salmonella* spp. and *Campylobacter* spp. [9]. The poultry industry has traditionally implemented non-chemical (e.g., physical removal of solids prior to the scalding step) and chemical interventions (e.g., chlorine and peroxyacetic acid rinses) against *Salmonella* spp. and *Campylobacter* spp., based on plant-to-plant experiences and word-of-mouth information shared among the industry. The validation of each intervention has been evaluated using traditional prevalence microbiological methods for *Salmonella* spp. and *Campylobacter* spp., which typically compares such prevalence before and after a particular intervention or a series of interventions is applied.

Typical chemical interventions that poultry processors utilize during first processing (e.g., evisceration) and second processing (e.g., deboning) include the use of sodium hypochlorite (chlorine) [11] and peroxyacetic acid (PAA) in equipment rinses, belt washers, inside-outside bird washers (IOBWs), on-line reprocessing (OLR) cabinets, pre-chillers, main chillers, shower heads, and dips/sprays. These chemicals and any chemical used as antimicrobial intervention in a federally inspected establishment must be listed under the USDA-FSIS safe and suitable ingredients used in production of meat, poultry, and egg products [12]. For instance, for PAA the maximum approved concentration is 2000 parts per million (ppm). Many chemical interventions have been studied for raw poultry products, and these must be approved for industry applications. Typically, laboratory validations are conducted to prove efficacy prior to field tests and/or application and chemicals should show at least, as a general rule, a 1 log CFU/mL reduction after the intervention application to be considered useful [13].

The use of PAA has increased in popularity among poultry processors, and research studies show its efficacy is greater than chlorine as well as other antimicrobials available for the poultry industry [14]. However, PAA has been associated with occupational concerns because of its corrosive and irritating effect on eyes, nasal passages, and skin [15]. OSHA has yet to establish occupational exposure limits for PAA; however, the American Conference of Governmental Industrial Hygienists (ACGIH) established an occupational exposure limit of 0.4 ppm as a short-term exposure limit for inhalable fraction and vapor [16]. Processors have been increasing PAA concentration levels at more locations to ensure compliance to regulatory standards; therefore, there is a need to re-assess the strategic use of PAA as an intervention in poultry processing to address occupational concerns and enhance microbial performance. The FSIS reported that between July 2020 and June 2021, the prevalence in raw chicken carcasses for *Salmonella* spp. was 3.42% (down slightly from the previous year) and for *Campylobacter* spp. was 16.45% (down significantly from the previous year). Similarly, the prevalence in raw chicken parts for *Salmonella* spp. was 6.53% (down from the previous year) and for *Campylobacter* spp. was 15.12% (down from the previous year) [17].

Despite poultry processors using a multi-hurdle approach to achieve the USDA-FSIS performance standards, there is minimal information regarding enumeration of *Salmonella* spp. and *Campylobacter* spp. levels in comparing individual chemical interventions or the contribution of these interventions in the multi-hurdle approach. This is the first biomapping study that incorporates ten sampling locations throughout carcass cleaning, evisceration, chilling, and deboning of chicken parts in comparing the microbial performance when all chemical interventions are turned on (normal chemical) versus the performance when the chemical interventions are turned off or reduced to the minimum allowed concentration (reduced chemical). The evaluation included indicator organisms, such as aerobic plate counts (AC) and Enterobacteriaceae (EB), as well as *Salmonella* spp. counts and *Campylobacter* spp. counts. Statistical process control parameters for each processing scheme and location were developed to assist the facility in continuous improvement of their food-safety system.

## 2. Materials and Methods

### 2.1. Sample Collection

The study was conducted on a commercial processing facility that processes on average 336,000 birds and runs in two lines at 175 birds per minute in the southern region of the United States. Samples were collected by trained plant personnel throughout a 25-month period of operations to account for flock-to-flock variability and day-to-day process variability. Whole chicken carcass and part rinses from a small birds (target 4.5 lb. live bird weight) were collected using 400 mL of buffered peptone water (BPW), (Millipore Sigma, Danvers, MA, USA). Rinses were immediately chilled and shipped overnight to the International Center for Food Industry Excellence (ICFIE) Food Microbiology laboratory at Texas Tech University for microbiological analysis.

### 2.2. Intervention Parameters

**Figure 1:**
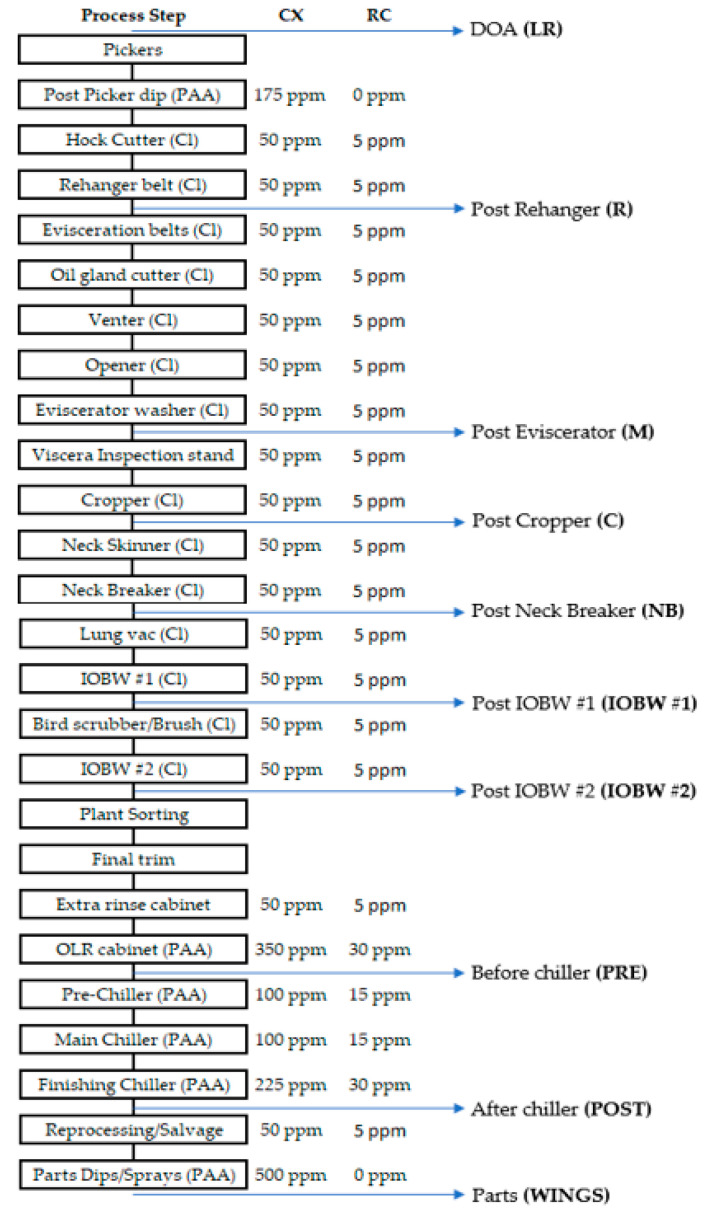
General flow chart of the process, identifying the CX and the RC processing schemes along with the sampling locations.

The normal processing conditions included chicken carcasses undergoing the standardized processing conditions of the operation with high levels of chemical interventions (CX—chemical treatments), including PAA, PAA + sodium hydroxide, and sodium hypochlorite, at various steps in the evisceration, chilling, and deboning processes, respectively. The reduced chemical treatment was planned to include no chemical interventions (just water) or reduced targeted chemical levels (RC—low chemical). The normal process interventions (CX) typically range from 100–400 ppm of PAA (in some cases in combination with sodium hydroxide to elevate the pH of the medium) and up to 50 ppm of total chlorine (sodium hypochlorite). For the low-chemical intervention process (RC), the chemical application was eliminated in several locations except for where needed as per the validated HACCP being verified by FSIS in the Public Health Information System (PHIS). Figure 1 shows a general flow chart of the process, identifying the CX and the RC processes and chemical concentrations along with the sampling locations. Ten locations throughout the processing line were sampled, including live receiving (LR)—where a warm and intact recently identified dead-on-arrival (DOA) was collected as the closest location to the actual live receiving step; rehanger (R); post eviscerator (M); post cropper (C); post neck breaker (NB); post inside-outside bird washer 1 (IOBW #1); post inside-outside bird washer 2 (IOBW #2); pre chilling (PRE); post chilling (POST); and parts (wings). At each location, at least ten rinses were taken per repetition for CX and RC treatments, five per shift. A total of 1309 samples were analyzed during the current study.

### 2.3. Microbial Indicators and Campylobacter *spp.* Enumeration

Rinses were homogenized by hand, and then, the TEMPO system (BioMérieux, Paris, France) was used for the enumeration of indicator microorganisms as well as *Campylobacter* spp. For aerobic plate counts (AC), the Association of Official Agricultural Chemists (AOAC) 121.204 was used, where TEMPO cards were incubated for 22–28 h at 35 ± 1 °C. For *Enterobacteriaceae* enumeration, the AOAC 050801 was used, where TEMPO cards were incubated for 22–28 h at 35 ± 1 °C. For *Campylobacter* spp. enumeration, the ISO 16140/AFNOR method was followed, where TEMPO cards were incubated for 44–48 h at 42 ± 1 °C under microaerophilic conditions using a gas pack generating system.

### 2.4. Salmonella *spp.* Enumeration and Prevalence

Rinses were homogenized by hand, and then, 30 mL of the rinses were combined with 30 mL of SalQuant solution (Hygiena, Camarillo, CA, USA). Samples were immediately incubated at 42 °C for 6 h for recovery. After incubation, the AOAC 081201 protocol for enumeration of *Salmonella* spp. using the BAX^®^ System SalQuant™ (Hygiena, Camarillo, CA, USA) was followed. Subsequent to enumeration, samples were placed again in an incubator at 42 °C for 18 h for enrichment. After incubation, if samples were not positive for BAX^®^ System SalQuant™, the BAX^®^ System RT-*Salmonella* Assay for detection was followed.

### 2.5. Statistical Analysis

All data were analyzed using R (Version 4.04) statistical analysis software to evaluate the difference in reduction of microbial loads after following the normal process interventions when compared to low-chemical process interventions on each of the 10 locations analyzed. All counts were transformed to log CFU/mL of rinse with exception of *Salmonella* spp. counts, which were reported as log CFU/sample (Log CFU/400 mL), and a *t*-test was performed to compare the counts at each location with normal process interventions and low chemical process interventions. If parametric assumptions were not met, the Wilcoxon Sum Rank Test or Mann–Whitney test was used as a non-parametric alternative for the *t*-test. A *p*-value of 0.05 or less was used to determine significant differences.

## 3. Results

The log CFU/mL (or log CFU/Sample for *Salmonella* spp. counts) reductions from live receiving to rehanger locations were significant for all testing conducted on indicator and pathogen bacteria. For indicator organisms, the average reduction for AC was 2.92 log CFU/mL (*p*-value < 0.001) and 2.41 log CFU/mL (*p*-value < 0.001) for the CX and RC treatments, respectively, while for EB the average reduction was 2.43 log CFU/mL (*p*-value < 0.001) and 2.29 log CFU/mL (*p*-value < 0.001) for the CX and RC treatments, respectively.

For pathogen enumeration, the average reduction from live receiving to rehanger locations for *Campylobacter* spp. was 3.18 log CFU/mL (*p*-value < 0.001) and 3.23 log CFU/mL (*p*-value < 0.001) for the CX and RC treatments, respectively, while for *Salmonella* spp., the average reduction was 2.27 log CFU/mL (*p*-value < 0.001) and 1.94 log CFU/mL (*p*-value < 0.001) for the CX and RC treatments, respectively.

In the nine locations following the live receiving (LR) location, for indicators and pathogens enumeration, the variation of the data points for the low-chemical treatment (RC) treatments was greater than those for the normal chemical treatment (CX) treatments.

For each of the sampling locations and all indicators as well as pathogens counts, the standard error (SE) was calculated to show dispersion of sample means around the population mean. The mean plus three standard error of the mean (mean + 3SE) was also calculated in each treatment to show the upper control limit per the USDA FSIS recommendation on statistical process control [18].

### 3.1. Aerobic Plate Counts (AC)

**Figure 2:**
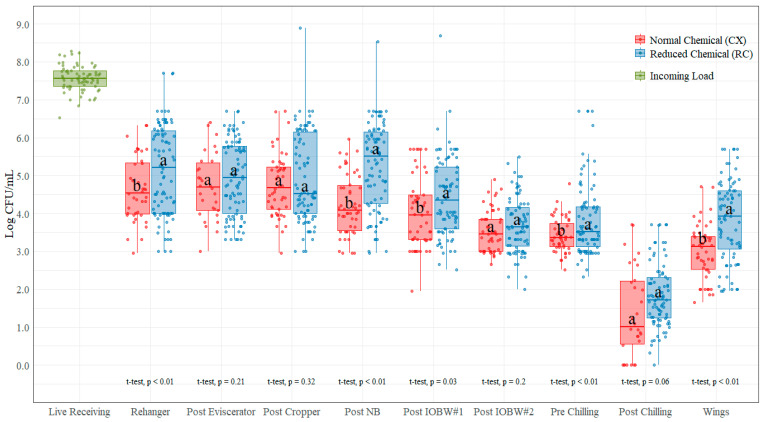
Aerobic plate counts (log CFU/mL) on each of the ten locations during the evisceration process under normal process interventions (CX) and lo- chemical process interventions (RC) on chicken rinses. In each boxplot, the horizontal line crossing the box represents the median, the top and bottom lines of the box represent the lower (0.25) and upper (0.75) quartiles, the vertical top lines represent 1.5 times the interquartile range, and the vertical bottom line represents 1.5 times the lower interquartile range. The dots represent the actual data points. ^a,b^ For each location, boxes with different letters are significantly different between treatments according to *t*-test analysis at *p*-value < 0.05.

The average incoming AC count measured at the live receiving area was 7.56 log CFU/mL (Table 1). These counts were prior to any (chemical) antimicrobial treatment at the processing plant. Subsequently, only feather removal and scalding, after hanging, stunning, and killing steps, were applied. There was a significant reduction from live receiving (7.56 CFU/mL) to rehanger location for both treatments: 4.64 log CFU/mL (CX with a *p*-value < 0.001) and 5.16 log CFU/mL (RC with a *p*-value < 0.001). The AC counts were not statistically different (*p* > 0.05) between CX and RC treatments at post-evisceration, post-cropper, post-IOBW #2, and post-chilling locations. Counts at the post rehanger, post neck breaker, post IOBW #1, pre chilling, and parts (wings) showed a statistically significant difference between treatments (*p* < 0.05), with the highest mean difference between treatments at the post-chilling location (0.45 log CFU/mL) and the lowest at the post-IOBW #2 location (0.15 log CFU/mL). For all locations, the low-chemical process (RC) shows greater counts than the normal process (CX) (see Figure 2). There was an increase in counts for both treatments from post-chilling to the parts (wings) location, where the CX treatment showed an average increase of 1.62 log CFU/mL; the RC treatment average increase was 2.01 log CFU/mL.

### 3.2. Enterobacteriaceae (EB)

**Figure 3:**
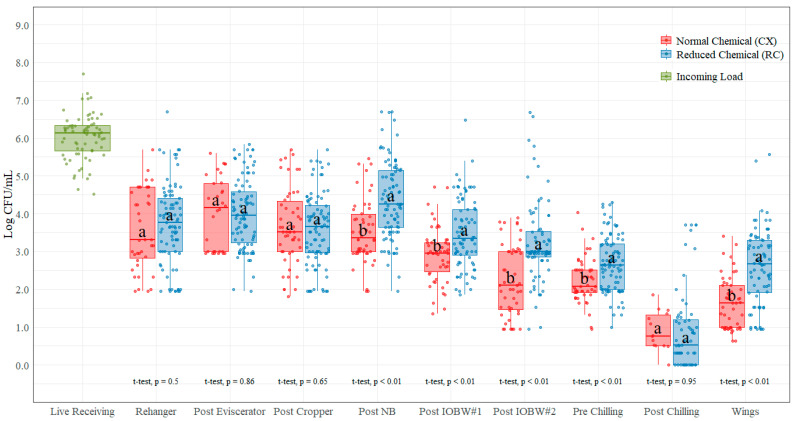
Enterobacteriaceae counts (log CFU/mL) on each of the ten locations during the evisceration process under normal process interventions (CX) and low-chemical process interventions (RC) on chicken rinses. In each boxplot, the horizontal line crossing the box represents the median, the top and bottom lines of the box represent the lower (0.25) and upper (0.75) quartiles, the vertical top lines represent 1.5 times the interquartile range, and the vertical bottom line represents 1.5 times the lower interquartile range. The dots represent the actual data points. ^a,b^ For each location, boxes with different letters are significantly different between treatments according to *t*-test analysis at *p*-value < 0.05.

The average incoming EB count measured at the live hanging area was 6.03 log CFU/mL (Table 2 and Figure 3). These counts were prior to any (chemical) antimicrobial treatment at the processing plant. The counts at the post-neck-breaker, post-IOBW #1, post-IOBW #2, pre-chilling. and parts (wings) locations had significant statistical differences (*p* < 0.05) between the CX and RC treatments, with the highest mean difference at the post-IOBW #2 location (1.01 log CFU/mL) and the lowest at the pre-chilling location (0.45 log CFU/mL). All locations showed higher counts with the RC treatments except for the post-evisceration and the post-cropper locations, where the RC treatment was lower than the CX treatment, with a mean difference of 0.04 log CFU/mL and 0.08 log CFU/mL, respectively. For the post-rehanger, post-evisceration, post-cropper, and post-chilling locations, there were no significant statistical differences (*p* > 0.05) between the CX and RC treatments.

### 3.3. Salmonella Detection and Enumeration

*Salmonella* spp. counts were substantially low when analyzed on a per-mL basis; thus, when transformed to log CFU/mL, some counts resulted in negative values (2.91% of the data with the CX treatment and 8.28% of the data with the RC treatment), making analysis and visualization more difficult for interpretation. Therefore, all data were transformed from to log CFU/sample equivalent to log CFU/400 mL to facilitate data visualization. The limit of quantification for SalQuant (LOQ) is 1 CFU/mL, but counts can be extrapolated below LOQ, as counts are obtained from a regression equation provided by the methodology, the reason why a new LOQ was established as 1% of the real LOQ (0.01 CFU/mL or 0.6 Log CFU/sample). Samples showing as <0.6 log CFU/sample were reported as 50% of the new LOQ (0.3 log CFU/sample). The same value was applied for samples that were not quantifiable but found positive for prevalence analysis. Samples that were not quantifiable nor detected were reported as 0 log CFU/sample. A summary of the parameters used for the data analysis can be found in Table 3.

**Figure 4:**
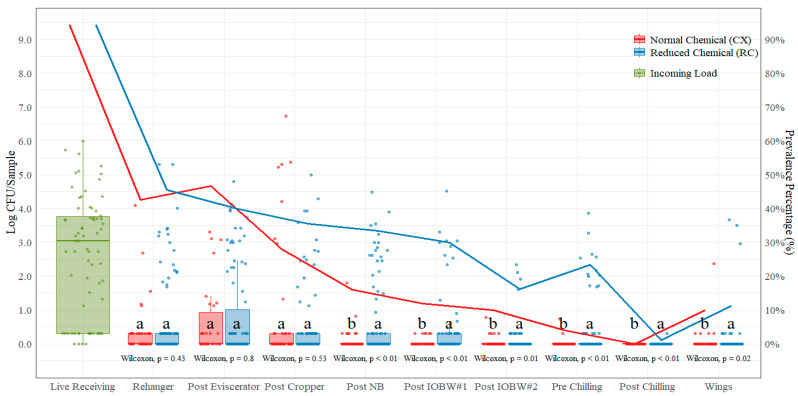
*Salmonella* spp. counts (log CFU/Sample) and prevalence (shown as solid lines) comparison on each of the ten locations during the evisceration process under normal process interventions (CX) and low-chemical process interventions (RC) on chicken rinses. In each boxplot, the horizontal line crossing the box represents the median, the top and bottom lines of the box represent the lower (0.25) and upper (0.75) quartiles, the vertical top lines represent 1.5 times the interquartile range, and the vertical bottom line represents 1.5 times the lower interquartile range. The dots represent the actual data points. ^a,b^ For each location, boxes with different letters are significantly different between treatments according to Wilcoxon test analysis at *p*-value < 0.05.

The average incoming *Salmonella* spp. count measured at the live hanging area was 2.63 log CFU/sample (Table 4). These counts were prior to any (chemical) antimicrobial treatment at the processing plant. Counts were statistically different (*p* < 0.05) between treatments in all sampling locations except for the post-evisceration and post-cropper locations. The RC treatment had greater counts at each sampling location except for the post-cropper location, where the lowest average count was at the RC treatment (0.67 log CFU/Sample). The highest average difference between CX and RC treatments was at the post-neck-breaker location (0.61 log CFU/sample) and the lowest at the post-chilling location (0.01 log CFU/sample). In addition to enumeration (counts), prevalence (Table 5) was performed on non-quantifiable samples using BAX^®^ system Real-Time *Salmonella* assays, and values are shown in Figure 4. The prevalence under the CX treatment is lower for all sampling locations except at the post-evisceration location.

### 3.4. Campylobacter *spp.*

**Figure 5:**
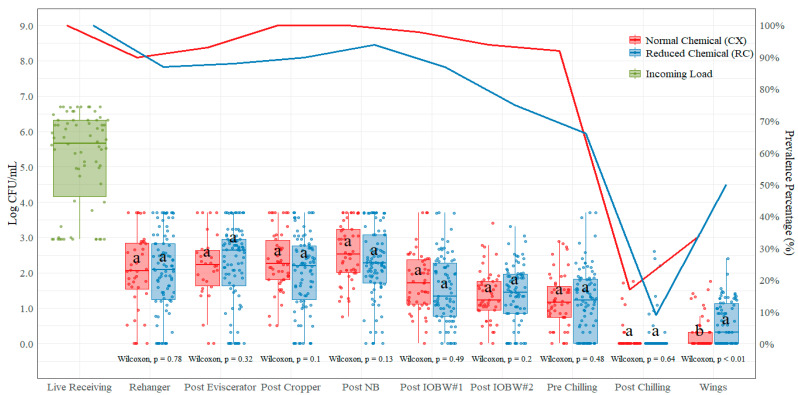
*Campylobacter* spp. counts (log CFU/mL) and prevalence (shown as solid lines) comparison on each of the ten locations during the evisceration process under normal process interventions (CX) and low-chemical process interventions (RC) on chicken rinses. In each boxplot, the horizontal line crossing the box represents the median, the top and bottom lines of the box represent the lower (0.25) and upper (0.75) quartiles, the vertical top lines represent 1.5 times the interquartile range, and the vertical bottom line represents 1.5 times the lower interquartile range. The dots represent the actual data points. ^a,b^ For each location, boxes with different letters are significantly different between treatments according to *t*-test analysis at *p*-value < 0.05.

The average incoming *Campylobacter* spp. count measured at the live hanging area was 5.23 log CFU/mL (Table 6). These counts were prior to any (chemical) antimicrobial treatment at the processing plant. The only location with significant mean difference (*p* < 0.05) between CX and RC treatments was the parts (wings) location, where the difference between treatments was 0.30 log CFU/mL (CX treatment with lower counts than the RC treatment). However, higher counts were shown in the CX treatments for post-rehanger (2.05 log CFU/mL), post-cropper (2.34 log CFU/mL), post-neck-breaker (2.57 log CFU/mL), post-IOBW #1 (1.75 log CFU/mL), pre-chilling (1.23 log CFU/mL), and post-chilling (0.18 log CFU/mL) locations (Figure 5). The highest mean difference between treatments was shown at the post-cropper location (0.34 log CFU/mL higher on the CX treatment) and the lowest at the post-rehanger and post-chilling locations (0.05 log CFU/mL on both locations, higher on the CX treatment).

Prevalence was obtained from the TEMPO^®^ quantification data, and values are shown in Table 7. The *Campylobacter* spp. incoming load measured at live receiving was 100.00% positive. After the slaughtering, bleeding, and defeathering (including scalding and picking) processing steps, the prevalence of *Salmonella* spp. was reduced to 90.00% (CX) and 86.70% (RC) positive, which represents a 10.00% (CX)/13.30% (RC) reduction without any chemical intervention applied other than under the RC treatment, where in some of the samples, the post-picker dip was kept at 175 ppm (PAA). After the rehanger, there was not a gradual reduction on counts; instead, the prevalence increased slightly from rehanger to the post-eviscerator location with both treatments: 93.33% positive with the CX treatment and 86.70% positive with the RC treatment. Furthermore, from the post-eviscerator to the post-cropper location, there was also an increase in prevalence with both treatments: 100.00% positive with the CX treatment and 90.00% positive with the RC treatment. At the post-neck-breaker location, with the CX treatment, the *Campylobacter* spp. prevalence stayed the same at 100% and with the RC treatment increased to 94.40%. There was a decrease in prevalence from the post-NB to the post-IOBW#1 location, and from the post-IOBW#1 to the post-IOBW#2 locations, *Campylobacter* spp. prevalence decreased from 98.00% to 94.00% positive with the CX treatment and from 86.90% to 75.30% positive with the RC treatment. There was also a decrease from the post-IOBW#2 (94.00% positive with CX and 75.30% with RC) and the pre-chilling location (92.00% positive with CX and 66.30% with RC).

## 4. Discussion

As observed in previous studies, the prevalence of *Salmonella* spp. was reduced from the pre-scalding to the post-chiller stages. These reductions were attributed to sequential washes and antimicrobial interventions applied during evisceration and in the pre- and post-chiller tanks [14,19,20,21,22]. Most of the research studies conducted on *Salmonella* spp. and *Campylobacter* spp. in poultry focus the microbiological methods on prevalence (%), whereas in the current study, we evaluated the quantification of indicator bacteria as well as pathogens (*Salmonella* spp. and *Campylobacter* spp.) in a processing operation running with chemical interventions and low levels of interventions, which makes the current research study unique. The sampling collection also occurred over a period of twenty-five months, capturing variability of flocks sampled and seasonality.

The significant log reductions from live receiving to the rehanger location for both indicator and pathogen loads provide validation data indicating that the scalding (washing effect and high temperature) and picking processes are key steps in bacterial reduction during poultry processing and a major pathogenic reduction stage for pathogen control if properly managed. The sample collected at the live receiving location included feathers, head, and feet, as well as any filth from the field, compared to the picked (plucked) bird at the rehanger location, where the feathers, head, and feet have been removed. As mentioned in previous studies [23], in general for industry professionals, a pathogen reduction of at least one logarithmic cycle from location to location is necessary to consider an intervention effective. In the current study, the average reduction from live receiving to rehanger across both treatments was 2.66 log CFU/mL (APC), 2.36 log CFU/mL (EB), 3.20 log CFU/mL (*Campylobacter* spp.), and 2.15 log CFU/sample (*Salmonella* spp.). At this particular processing plant, there is no chemical treatment applied in the scalding or the defeathering process. As indicated earlier, there is a post-picker dip with up to 175 ppm of PAA immediately after the last picker, which showed to be statistically significant when comparing CX and RC treatments for AC and *Salmonella* spp. counts. Therefore, even without any pH adjustment treatment in the scalder tanks (one of the common antimicrobial interventions used in the poultry industry), the softening and removal of the feathers while keeping the bird warm during this process are definitely an important aid in bacterial reduction for the process.

The need to optimize the rather widespread use of PAA as interventions throughout the process is critical due to concerns on dose and time of contact variability [10] and the occupational concerns mentioned earlier [15]. Therefore, the current research study provides a standardized methodology to generate the evidence needed for the identification of focused intervention locations in the process, more specifically the use of PAA, in selected locations within first and second processing to maximize the efficacy and improved the microbial performance of the process.

In another study, it was determined that reductions in the AC and EB counts were not consistent between the post-scalding and post-defeathering locations [24] and did not provide a clear indication of what microorganisms could be affecting those results. We learned that the reduction from the live receiving to the rehanger location under the CX treatment on both AC (2.92 Log CFU/mL) and EB (2.43 Log CFU/mL) was consistent, and the counts remained somewhat constant between the rehanger and the post-neck-breaker location, suggesting that up to the post-neck-breaker location, there is no major reduction on AC and EB counts even with high levels of chemical interventions applied. In fact, the post-evisceration and post-cropper locations showed no significant statistical difference between the CX and RC treatments (*p* > 0.05).

Poultry processors have implemented various antimicrobial interventions to reduce cross contamination and minimize the presence of foodborne pathogens, such as *Salmonella* spp. and *Campylobacter* spp., during poultry processing. However, limited information on comprehensive biomapping conducted at a commercial poultry processing facility—which included enumeration of pathogens as well as prevalence—is available in the literature. Limited research studies are available, such as those using chicken parts, conducted in laboratory settings and in controlled environments. In the current study, whole birds and parts (wings) samples were collected over the course of twenty-five months and included quantification of indicators and pathogens in a plant setting, therefore making the current bio-mapping more representative of the process variability and allowing this processor to establish a facility-specific microbial baseline for decision making on the intervention’s effectiveness.

The processing facility where the current research study was conducted is operating under the New Poultry Inspection System (NPIS) and has a line-speed waiver to process in evisceration, at line speeds of up to 175 birds per minute (BPM). The multi-hurdle approach for antimicrobial interventions at this processing facility, whether under the CX or the RC treatments, achieved post-chill pathogen counts of less than 0.27 and 0.57 log CFU/mL (*Campylobacter* spp.) or 0.07 and 0.15 log CFU/Sample (*Salmonella* spp.), respectively. These levels, according to the risk assessments of *Salmonella* spp. in broiler chickens [25], have a very low probability for causing illness, without even considering the effect of thermal processing on risk reduction from the raw poultry carcass or part evaluated. Furthermore, when comparing these results at the parts location (wings), the levels are below 1 log CFU/mL (*Campylobacter* spp.) or 1 log CFU/Sample (*Salmonella* spp.), which also represents a very low probability of illness. Therefore, the current data suggest that the increased evisceration line speed under NPIS does not affect or increase the risk of illness caused by foodborne pathogens, such as *Salmonella* spp. and *Campylobacter* spp. [26].

### 4.1. Aerobic Counts (AC)

There were significant statistical differences between CX and RC treatments observed at the rehanger location (0.51 CFU/mL with lower counts shown with the CX treatment). These results suggest that the use of the post-picker dip, located immediately after the last picker and containing up to 250 ppm PAA, may have an improved effect in the overall process for pathogen control. There was no statistical difference between treatments at the post-evisceration and post-cropper locations, indicating that neither the chlorinated washer located immediately after the removal of viscera from the birds nor the washer and brushes removing crops from the probes may have a reduction effect in the aerobic counts.

At the NB location, there was a statistically significant reduction in counts with the CX treatment, while the counts with the RC treatment appeared to increase. This suggests that chemical interventions are needed at this location to ensure proper sanitizing of the neck-breaker blades to reduce cross contamination. Because the birds are hung upside down, all the fluids draining from the cavity of the birds pass through the neck area. At this step of the process, the release of these fluids when breaking the necks may require a chemical treatment to reduce the AC load. Furthermore, the very next processing step, the first inside-outside bird washer, seems to have a beneficial effect when chemical interventions are used in reducing aerobic counts. The average reduction at the post-IOBW #1 location from the previous steps (excluding live receiving) was 0.55 log CFU/mL (CX treatment) and 0.64 log CFU/mL (RC treatment).

The brushes (after the IOBW #1) and the subsequent IOBW #2 seem to not have a major effect in AC levels with the addition of chemicals at those two steps, as there are no statistical differences between CX and RC treatments at the post-IOBW #2 location. This could be due to the reduction already achieved by the IOBW #1. However, at the pre-chilling location, which is after the on-line reprocessing (OLR) cabinet, there is a significant statistical difference between CX and RC treatment; however, the reduction is only 0.11 log CFU/mL. There was also an increase for the RC treatment from the post-IOBW#2 to the pre-chilling location (0.15 log CFU/mL). This suggests that the chemical effect at the OLR applied in this facility may not be an important antimicrobial intervention in the AC reduction and will need to be optimized. The typical chemical used at this location is PAA, at concentrations ranging from 300 ppm to 400 ppm under normal processing.

At the post-chill location, the difference between CX and RC treatments is not statistically significant, with a 2.04 log CFU/mL reduction from pre-chill to post-chill locations under the CX treatment and 2.00 log CFU/mL under the RC treatment. The lowest AC counts with both treatments occurred at the post-chilling location (lower with the CX treatment), indicating that the temperature reductions and chemical treatments in the pre-chiller, main-chiller, and post-chiller when combined are effective for reducing AC counts.

There is also a significant statistical difference between treatments at the parts location (wings), with CX treatment at 0.84 log CFU/mL, lower in average than the RC treatment. The overall reduction at this location has been previously reported at 1.27 log CFU/mL on a laboratory spray application setting on breast fillets [27]. Parts dips have become popular in commercial processing facilities, and they are currently widely used in the poultry industry, with concentrations of PAA up to 400 ppm to help in complying with parts performance standards. This antimicrobial intervention has proven to be very effective in reducing the loads of AC, as shown in the current research study.

### 4.2. Enterobacteriaceae Counts (EB)

Similar to what was found with the AC counts, the EB counts at the post-evisceration and post-cropper locations were not significantly different between the CX and RC treatments. In addition to these locations, the post-rehanger EB counts were also not significantly different between the treatments. However, there is a significant difference between the treatments at the post-neck-breaker location, with the RC treatment being higher than the CX treatment, on average 0.84 log CFU/mL. This difference could be due to the antimicrobial effect of the neck-breaker equipment washers. The use of chemicals during the process seem to have a positive impact when measuring EB at the post-neck-breaker location, which along with the removal of the viscera and crops, creates an opening around the neck area, helping drainage of contamination during processing. However, there was not much change among the counts from the rehanger to the neck-breaker locations, with an average EB count of 3.71 log CFU/mL (CX) and 3.92 log CFU/mL (RC) in these four locations. As mentioned in previous studies, certain steps, such as those within the evisceration process, may contribute to higher levels of contamination [28], and in the current research study, we found that the EB counts do not seem to change much across these locations.

The chemical usage in the IOBWs as well as in the brushes between the washers seem to also have a positive impact in reducing EB counts, which is displayed in the significant differences between the treatments at these locations. Whereas no significant statistical differences were found at post-chill location, indicating that not much effect on EB was accomplished by the use of chemical in the chilling system, the lower temperature in the system may have a positive impact in the reduction of EB counts between pre-chill and post-chill locations. There is a significant statistical difference between treatments at the pre-chilling and parts (wings) locations, which reinforces the findings that parts dips with PAA have a positive impact in bacterial reduction in skin-on part samples.

### 4.3. Salmonella *spp.*

There was a statistical difference in *Salmonella* spp. counts between treatments at each sampling location (except for the post-evisceration and post-cropper locations), with CX being the lowest at each sampling location with the exception of post-cropper, where the CX treatment was lower (0.67 log CFU/mL at RC vs. 0.75 log CFU/mL at CX). The pattern for prevalence was very similar, with the highest prevalence of *Salmonella* spp. under the RC treatments except for samples collected at the post-evisceration location. At this location, the CX treatment had a slightly higher prevalence than the RC treatment.

The largest average difference between treatments was at the post-neck-breaker location, validating that cross-contamination control and adequate sanitary dressing in neck breaking are key steps in the reduction of *Salmonella* spp. Furthermore, chilling (pre main and post chiller) continued to be a crucial step in microbial control during poultry processing, which is validated by the 0% (CX) and 0.94% (RC) prevalence at the post-chill location, significantly lower than the performance standard limits.

The reduction in prevalence from the live receiving (>90%) to the rehanger (~40%) follows the same trend as with the quantification reduction at these two locations. Even though the prevalence reduction is close to 50%, in quantification, the average reduction from live receiving to rehanger locations (2.27 log CFU/Sample for CX and 1.94 log CFU/Sample for RC) was higher than 90% with the CX treatment and 75% with the RC treatment, and it can only be seen with quantification data. These discrepancies are a confirmation than prevalence alone is not a good indicator of food safety [29].

### 4.4. Campylobacter *spp.*

After the live receiving location, all locations except for parts (wings) show no significant difference between treatments CX and RC. Only the parts (wings) location, with an average difference of 0.30 log CFU/mL, showed minimal effect under the CX treatment, which is consistent with the AC and EB indicators as well as *Salmonella* spp. loads. This provides some evidence that parts interventions are effective in reducing pathogen loads. As previously reported, the use of antimicrobial interventions, such as post-chilling immersion tanks or spraying systems using high concentration of chemicals (with short contact times), have proven to be an added hurdle after primary chilling that further facilitates the reduction of pathogens on poultry carcasses [10].

As seen on the results, after the live receiving location, there seemed to be not much change in counts from the rehanger to the post-neck-breaker locations, which is a pattern observed with AC and EB counts. However, there is a reduction at the first IOBW #1, showing an average 0.82 log CFU/mL (CX) and 0.71 log CFU/mL (RC) from the previous location. Furthermore, between the post IOBW #1 and the pre-chilling locations, there is not much change in *Campylobacter* spp. counts until the post-chilling location. This provides strong evidence that the chilling of the birds is the primary step in pathogen reduction.

Prevalence of *Campylobacter* spp. under the CX treatment remains constant between 90% and 100% through the pre-chilling location; however, as discussed before, there is a 3.18 log CFU/mL reduction from live receiving to the rehanger location. This reduction is negligible when only looking at prevalence. Similarly, under the RC treatment, the prevalence of *Campylobacter* spp. remains between 85% and 100% through the post-IOBW #1 location, disregarding the reduction in counts from live receiving to rehanger of 3.23 log CFU/mL, which is a strong evidence that prevalence alone cannot be used as a sole representation of the microbial loads within a poultry-processing facility [29].

## 5. Conclusions

Pathogen quantification can result in improved risk assessment where chemical interventions can be targeted to stages with higher indicator and pathogen bacteria counts. The current research study provides evidence for the application of chemical treatments in strategic locations during poultry processing rather than the use of an array of interventions at different locations, thus assisting the processor to customize their antimicrobial intervention regimes and focus these efforts in higher-risk areas.

The development of biomapping baselines that include quantification of pathogens leads to the development of statistical process control parameters to support food safety management decision making. Nonparametric statistical process control can be approached to more representatively use pathogen prevalence and quantification data together, resulting in more educated decisions than using exclusively prevalence data.

In the current research study, it was evident that the scalding and picking processing steps leading up to the evisceration process are key steps in the reduction of indicator and pathogen bacteria. Furthermore, the reduction achieved between live receiving and rehanger is almost constant for both treatments (CX and RC) for any of the indicator and pathogen bacteria tested up to the neck-breaker location. After such step, the incorporation of chemicals (e.g., sodium hypochlorite) at the first inside-outside bird washer (IOBW #1), along with good sanitary dressing practices, seem to have the best performance. Therefore, the first step in the evisceration process that needs to have chemicals based on the results of the current study is the IOBW #1.

The on-line reprocessing (OLR) cabinet does not seem to have a major impact in bacterial reduction in this operation with either CX or the RC treatments; however, the chilling system, including the pre chiller, main chiller, and post-finishing chiller, were shown to be a major contributor to pathogen reductions (combining low water temperature and chemical usage, such as PAA) for bacterial reduction, thus indicating that the chiller process should be optimized as the second main location for chemical application in the process. The final antimicrobial intervention step, shown in the current study to have an impactful bacterial-reduction performance, is the parts dips, where PAA is mostly used.

The data generated from the current study demonstrate that the use of *Salmonella* spp. or *Campylobacter* spp. prevalence as a sole measurement of food-safety performance is not adequate or representative of the whole picture of contamination in a dynamic system. Pathogen prevalence is part of the equation, and several other variables, such as quantification, are necessary to make decisions that will improve the food-safety system’s performance. There have been models published identifying risk factors for *Salmonella* control in poultry-processing operations [29], which support the conclusions of the current study. Published risk assessments support this approach, and the results of the current study can be used to conduct probabilistic quantitative microbial risk assessments similar to those conducted in prior publications (QMRA) [30]. Finally, this integrated approach to measure the performance of the pathogen-control system provides a risk-based approach to food-safety management and therefore is needed to establish a new performance standard for *Salmonella* spp. and *Campylobacter* spp. that is based on loads. A better performance-standard system can contribute in a better way to help achieve the Healthy People 2030 goals [31,32].

There is a significant amount of data generated by research conducted by poultry processors, who collect far more microbiological data than the official sampling programs of the USDA-FSIS sampling plans (e.g., *Salmonella* spp. 52-rolling window—one sample per week). Federally inspected establishments collect on a routine basis samples before and after chilling for every 22,000 birds processed. For example, if a single evisceration line processes 660,000 birds in one week, there would be a total of thirty samples (30) collected in one week for one of the indicator organisms compared to one (1) sample collected by USDA FSIS. These samples are in addition to other microbial samples collected by each establishment to evaluate the performance of some of their intervention schemes. Furthermore, poultry processors, through biomapping sampling, select more significant sampling locations that better represent the microbiological performance of the process. With more repetitions and extra sampling locations, the poultry industry can generate sufficient quantitative data on pathogen loads that, when statistically analyzed, would serve as a better measurement for the establishment’s microbial performance and to generate actual risk-based performance standards. Therefore, it is important to consider outside data, such as that generated from the current research study, to evaluate large datasets from a variety of operations to establish a plant’s microbial performance [33].

## Figures and Tables

**Table 1:** Aerobic plate counts (log CFU/mL) on each of the ten locations during the evisceration process under normal process interventions (CX) and low-chemical process interventions (RC) on chicken rinses.

Location	Aerobic Plate Counts (Log CFU/mL)					
	Chemical (CX)			Reduced Chemical (RC)		
	Mean ± SE ^1^	Mean + 3SE	*n*	Mean ± SE	Mean + 3SE	*n*
Live Receiving ^2^	7.56 ± 0.04 ^a^	7.68	70	7.56 ± 0.04 ^a^	7.68	70
Rehanger	4.64 ± 0.14 ^b^	5.04	40	5.16 ± 0.13 ^bc^	5.53	90
Post Eviscerator	4.71 ± 0.16 ^b^	5.19	30	4.95 ± 0.11 ^bc^	5.27	90
Post Cropper	4.75 ± 0.12 ^b^	5.10	50	4.92 ± 0.12 ^c^	5.29	90
Post NB	4.22 ± 0.11 ^c^	4.56	50	5.25 ± 0.12 ^b^	5.61	90
Post IOBW#1	4.03 ± 0.14 ^c^	4.43	50	4.43 ± 0.12 ^d^	4.77	84
Post IOBW#2	3.54 ± 0.08 ^d^	3.77	50	3.68 ± 0.08 ^e^	3.92	89
Pre Chilling	3.42 ± 0.06 ^d^	3.61	50	3.84 ± 0.10 ^e^	4.14	98
Post Chilling	1.39 ± 0.19 ^f^	1.95	40	1.84 ± 0.08 ^f^	2.09	106
Parts (Wings)	3.01 ± 0.10 ^e^	3.31	50	3.84 ± 0.11 ^e^	4.18	92

^1^ Standard error of the mean; ^2^ For Live Receiving location, there was no treatment applied (CX nor RC); therefore, the same values are reported for each treatment on the table; ^a–f^ For each Location, with each treatment (CX and RC), Different Letters are Significantly Different according to ANOVA *p*-value < 0.01.

**Table 2:** Enterobacteriaceae counts (log CFU/mL) on each of the ten locations during the evisceration process under normal process interventions (CX) and low-chemical process interventions (RC) on chicken rinses.

Location	Enterobacteriaceae Counts (Log CFU/mL)					
	Chemical (CX)			Reduced Chemical (RC)		
	Mean ± SE ^1^	Mean + 3SE	*n*	Mean ± SE	Mean + 3SE	*n*
Live Receiving ^2^	6.03 ± 0.07 ^a^	6.25	70	6.03 ± 0.07 ^a^	6.25	70
Rehanger	3.60 ± 0.17 ^c^	4.10	40	3.74 ± 0.11 ^cd^	4.07	90
Post Eviscerator	4.04 ± 0.17 ^b^	4.56	30	4.00 ± 0.10 ^c^	4.30	90
Post Cropper	3.67 ± 0.15 ^bc^	4.10	50	3.59 ± 0.10 ^d^	3.89	90
Post NB	3.53 ± 0.12 ^c^	3.89	50	4.37 ± 0.11 ^b^	4.69	90
Post IOBW#1	2.91 ± 0.10 ^d^	3.22	50	3.48 ± 0.10 ^de^	3.78	84
Post IOBW#2	2.24 ± 0.13 ^e^	2.63	50	3.25 ± 0.11 ^e^	3.59	89
Pre Chilling	2.24 ± 0.08 ^e^	2.50	50	2.69 ± 0.08 ^f^	2.92	98
Post Chilling	0.90 ± 0.08 ^g^	1.15	40	0.92 ± 0.10 ^g^	1.23	106
Parts (Wings)	1.64 ± 0.10 ^f^	1.94	50	2.60 ± 0.11 ^f^	2.91	92

^1^ Standard error of the mean; ^2^ For Live Receiving location, there was no treatment applied (CX nor RC); therefore, the same values are reported for each treatment on the table; ^a–g^ For each Location, with each treatment (CX and RC), Different Letters are Significantly Different according to ANOVA *p*-value < 0.01.

**Table 3:** Observed and reported parameters established for *Salmonella* spp. quantification and prevalence analysis.

Observed SalQuant Result (Log CFU/Sample)	Observed Prevalence Result	Reported SalQuant Result (Log CFU/Sample)	Reported Prevalence Result
No Result	Negative	0	Negative
No Result	Positive	0.3	Positive
Less than 0.6	NA ^1^	0.3	Positive
More or equal than 0.6	NA	Observed SalQuant result	Positive

^1^ Not applicable, as prevalence test is not necessary in samples quantified and detected by SalQuant.

**Table 4:** *Salmonella* spp. counts (log CFU/sample) on each of the ten locations during the evisceration process under normal process interventions (CX) and low-chemical process interventions (RC) on chicken rinses.

Location	*Salmonella* spp. Counts (Log CFU/Sample)					
	Chemical (CX)			Reduced Chemical (RC)		
	Mean ± SE ^1^	Mean + 3SE	*n*	Mean ± SE	Mean + 3SE	*n*
Live Receiving ^2^	2.63 ± 0.21 ^a^	3.26	70	2.63 ± 0.21 ^a^	3.26	70
Rehanger	0.36 ± 0.13 ^bc^	0.74	40	0.69 ± 0.13 ^bc^	1.09	90
Post Eviscerator	0.63 ± 0.19 ^b^	1.21	30	0.79 ± 0.14 ^b^	1.21	90
Post Cropper	0.72 ± 0.24 ^bc^	1.44	50	0.57 ± 0.12 ^bc^	0.93	90
Post NB	0.09 ± 0.04 ^cd^	0.21	50	0.66 ± 0.12 ^bc^	1.03	90
Post IOBW#1	0.04 ± 0.01 ^d^	0.08	50	0.43 ± 0.11 ^bc^	0.75	84
Post IOBW#2	0.04 ± 0.02 ^d^	0.10	50	0.13 ± 0.05 ^bc^	0.27	89
Pre Chilling	0.02 ± 0.02 ^d^	0.07	50	0.34 ± 0.08 ^bc^	0.60	98
Post Chilling	0.00 ± 0.00 ^d^	0.00	40	0.00 ± 0.00 ^c^	0.00	106
Parts (Wings)	0.07 ± 0.05 ^d^	0.22	50	0.15 ± 0.07 ^bc^	0.35	92

^1^ Standard error of the mean; ^2^ For Live Receiving location, there was no treatment applied (CX nor RC); therefore, the same values are reported for each treatment on the table; ^a–d^ For each Location, with each treatment (CX and RC), Different Letters are Significantly Different according to Krustal–Wallis test at *p*-value < 0.01.

**Table 5:** Prevalence of *Salmonella* spp. at each Sampling Location for each Treatment: Normal Chemical (CX) and Reduced Chemical (RC).

Location	Prevalence (%)	
	Normal Chemical (CX)	Reduced Chemical (RC)
Live Receiving *	94.29%	94.29%
Rehanger	42.50%	45.60%
Post Eviscerator	46.70%	40.00%
Post Cropper	28.00%	35.60%
Post Neck Breaker	16.00%	33.30%
Post IOBW #1	12.00%	30.00%
Post IOBW #2	10.00%	16.20%
Pre Chilling	4.00%	23.33%
Post Chilling	0.00%	1.11%
Parts (Wings)	10.00%	11.20%

* Percentages are the same under CX and RC because at Live Receiving location, no chemical treatment was applied.

**Table 6:** *Campylobacter* spp. counts (log CFU/mL) on each of the ten locations during the evisceration process under normal process interventions (CX) and low-chemical process interventions (RC) on chicken rinses.

Location	*Campylobacter* spp. Counts (Log CFU/mL)					
	Chemical (CX)			Reduced Chemical (RC)		
	Mean ± SE ^1^	Mean + 3SE	*n*	Mean ± SE	Mean + 3SE	*n*
Live Receiving ^2^	5.23 ± 0.16 ^a^	5.72	70	5.23 ± 0.16 ^a^	5.72	70
Rehanger	2.05 ± 0.18 ^cd^	2.58	40	2.00 ± 0.12 ^bc^	2.37	90
Post Eviscerator	2.18 ± 0.18 ^c^	2.71	30	2.23 ± 0.12 ^b^	2.59	90
Post Cropper	2.34 ± 0.12 ^bc^	2.70	50	2.00 ± 0.11 ^bc^	2.33	90
Post NB	2.57 ± 0.12 ^b^	2.92	50	2.25 ± 0.11 ^b^	2.57	90
Post IOBW#1	1.75 ± 0.12 ^d^	2.10	50	1.54 ± 0.10 ^cd^	1.85	90
Post IOBW#2	1.36 ± 0.10 ^e^	1.67	50	1.38 ± 0.09 ^cd^	1.65	89
Pre Chilling	1.23 ± 0.11 ^e^	1.56	50	1.18 ± 0.10 ^d^	1.47	98
Post Chilling	0.18 ± 0.07 ^f^	0.39	40	0.13 ± 0.05 ^f^	0.27	106
Parts (Wings)	0.27 ± 0.07 ^f^	0.48	50	0.57 ± 0.06 ^e^	0.76	92

^1^ Standard error of the mean; ^2^ For Live Receiving location, there was no treatment applied (CX nor RC); therefore, the same values are reported for each treatment on the table; ^a–f^ For each Location, with each treatment (CX and RC), Different Letters are Significantly Different according to Krustal–Wallis test at *p*-value < 0.01.

**Table 7:** Prevalence of *Campylobacter* spp. at each Sampling Location for each Treatment: Normal Chemical (CX) and Reduced Chemical (RC).

Location	Prevalence (%)	
	Normal Chemical (CX)	Reduced Chemical (RC)
Live Receiving *	100.00%	100.00%
Rehanger	90.00%	86.70%
Post Eviscerator	93.33%	87.80%
Post Cropper	100.00%	90.00%
Post NB	100.00%	94.44%
Post IOBW#1	98.00%	86.90%
Post IOBW#2	94.00%	75.30%
Pre Chilling	92.00%	66.30%
Post Chilling	17.50%	9.43%
Parts (Wings)	34.00%	50.00%

* Percentages are the same under CX and RC because at Live Receiving location, no chemical treatment was applied.

## Data Availability

Data available on request from the corresponding author. The data are not publicly available due to privacy from the poultry-processing partner that allowed the project to be conducted within their poultry-processing facility.
